# Is Totally Tubeless Percutaneous Nephrolithotomy a Safe and Efficacious Option for Complex Stone Disease?

**DOI:** 10.3390/jcm13113261

**Published:** 2024-05-31

**Authors:** Nir Tomer, Vinay Durbhakula, Kavita Gupta, Raymond Khargi, Blair Gallante, William M. Atallah, Mantu Gupta

**Affiliations:** Department of Urology, Icahn School of Medicine at Mount Sinai, 425 W. 59th Street, Suite 4F, New York, NY 10019, USA; nir.tomer@mountsinai.org (N.T.);

**Keywords:** totally tubeless PCNL, safety, kidney stones

## Abstract

**Background**: Percutaneous nephrolithotomy is the gold standard treatment for large, complex intrarenal stones. Historically, this was performed using a nephrostomy tube (PCN) and/or internalized ureteral stent at the end of the procedure. However, totally tubeless nephrolithotomy (tt-PCNL) is a novel technique where no tubes (no stent nor nephrostomy tube) are left post-operatively. We review the literature on this subject regarding peri-operative outcomes, post-operative outcomes, and potential complications of the procedure, discuss our technique, and make recommendations on implementation for centers not currently utilizing the procedure. **Materials and methods**: We performed a comprehensive search of the literature on totally tubeless nephrolithotomy using MEDLINE database search. Our search included prior review articles, meta-analyses, systematic reviews, primary research articles, case reports, and case studies. **Results**: In comparison to prior approaches where a stent or nephrostomy tube is placed, tt-PCNL has a similar complication rate and better post-operative outcomes. Totally tubeless PCNL has similar operative times and similar changes in hemoglobin. However, it had shorter length of stays across all studies. The mean difference in length of stay in the studies reviewed was 1.96 days. Additionally, tt-PCNL had decreased post-operative analgesic requirements and pain scores. **Conclusions**: This review highlights totally tubeless percutaneous nephrolithotomy as a safe and feasible surgical technique with improved outcomes in properly selected patients.

## 1. Introduction

Kidney stones are a common urologic condition affecting one in nine individuals. According to the most recent NHANES analysis, the prevalence of kidney stones was 11% in the United States [1]. Treatment modality depends on the size, complexity, and location of the stone. When stones are >2 cm in size or are >1 cm and located in the lower pole, the American Urologic Association (AUA) guidelines recommend percutaneous nephrolithotomy (PCNL) as first line of therapy [2,3]. Percutaneous nephrolithotomy (PCNL) is a minimally invasive surgical procedure used to remove large and/or complex kidney stones [2,3]. When PCNL was first described in 1976, it was standard to leave a percutaneous nephrostomy tube (PCNT) post-operatively [4]. Leaving a nephrostomy tube was thought to ensure drainage of urine, clot, and residual stone fragments and provide hemostasis of the dilation tract [4,5]. However, leaving a nephrostomy tube was bothersome to the patient, and prolonged placement can lead to persistent urinary leakage from the flank. As a result, tubeless PCNL (t-PCNL) was introduced in the late 1990s, where an internalized double-J stent was left as an alternative method for post-operative drainage [4]. This was shown to reduce length of stay, analgesia requirements, post-operative pain, and cost [6]. Despite the improvements from the nephrostomy tubes, double-J stents can still lead to complications. Patients can suffer from stent colic and the removal can subject the patient to an additional procedure with its own risks [7,8]. As a result, totally tubeless PCNL (tt-PCNL) was developed whereby no external or internal drainage tubes are left behind, and the patient is rendered totally tubeless at the end of the procedure [8]. In this article, we review comparative and non-comparative studies on tt-PCNL, describe our approach, and provide recommendations for implementation. Additionally, we review the relevant percutaneous nephrolithotomy literature on ambulatory surgery, obesity, and hemostatic agents. 

## 2. Materials and Methods

**Figure 1:**
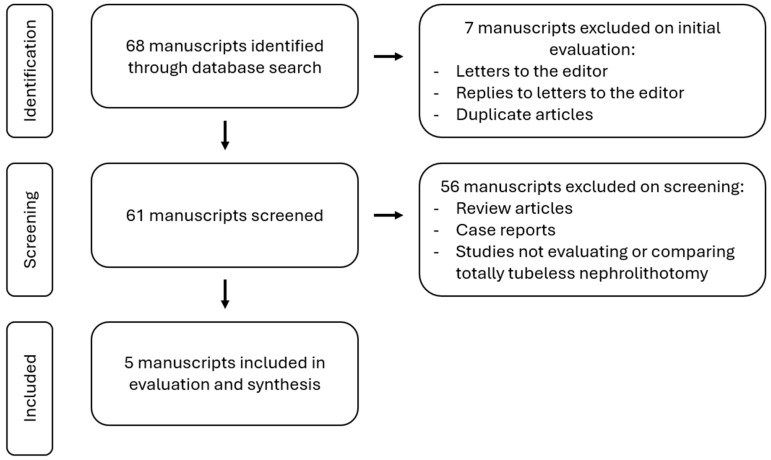
Flow diagram of search criteria and screening.

We performed a MEDLINE database search using the following terms: totally tubeless percutaneous nephrolithotomy, tubeless percutaneous nephrolithotomy, outcomes, and the MeSH term percutaneous nephrolithotomy. The articles found included articles dated from 1986 to 2023. A variety of articles were screened including prior review articles, meta-analyses, systematic reviews, primary research articles, case reports, and case studies. Ultimately, five studies were included that compared tt-PCNL to alternative techniques. Our search criteria and screening process are summarized in Figure 1. Across all five studies, 442 patients were included in the comparison. These five studies were evaluated and summarized as a narrative review. Table 1 summarizes the five studies and their methodology. 

## 3. Results

### 3.1. Comparison of Peri-Operative Outcomes

Totally tubeless PCNL has been described as a feasible option and peri-operative outcomes appear to be similar to those of tubeless and standard PCNL. The key peri-operative outcomes include operative time, blood loss, and length of stay (LOS). The peri-operative outcomes reported in the studies are summarized in Table 2. Blood loss or hemoglobin change does not appear to differ when compared to standard, tubeless, or totally tubeless PCNL [7,8,9,10,11]. Crook reported a Hb drop of 2.0 and 1.2 for t-PCNL and tt-PCNL, respectively (*p* > 0.05) [11]. Yun reported an EBL of 163 cc and 158 cc for standard PCNL and tt-PCNL, respectively (*p* = 0.45) [9]. Istanbulluoglu reported a Hb drop of 1.57, 1.40, and 1.62 for tt-PCNL, t-PCNL, and standard PCNL (*p* = 0.297) [8]. Moosanejad reported a Hb drop of 1.51 and 2.27 for standard PCNL and tt-PCNL (*p* = 0.58) [7]. Finally, Bhat reported a Hb drop of 1.01, 1.55, and 1.20 for standard PCNL, t-PCNL, and tt-PCNL (*p* = 0.07) [10]. Furthermore, operative time appears to be the same or shorter in tt-PCNL when compared to t-PCNL or standard PCNL [7,8,9,10,11]. Yun et al. and Istanbulluoglu et al. both found no difference in operative time [8,9]. Yun reported an operative time of 148.5 and 128.7 min for standard and tt-PCNL (*p* = 0.06) and Istanbulluoglu reported an operative time of 53.02, 51.29, and 60.07 min for tt-PCNL, t-PCNL, and standard PCNL (*p* = 0.130) [8,9]. However, Moosanejad et al. found that tt-PCNL had the shortest operative times. Moosanejad reported an operative time of 53.37 and 50.32 min for standard and tt-PCNL (*p* = 0.005) [7].

Importantly, all five studies that compared the PCNL surgical techniques found that the duration of hospital stay was shorter in the tt-PCNL cohort when compared to t-PCNL and standard PCNL [7,8,9,10,11]. The magnitude of difference varied between studies. The shortest reported LOS was in Moosanejad et al., who reported a mean LOS of 1.25 days in the tt-PCNL cohort and 2.95 days in the standard PCNL group [7]. Istanbulluoglu et al. reported a similar LOS difference [8]. The longest LOS was reported by Yun et al. who reported a mean LOS of 3.92 days in their tt-PCNL cohort and 8.25 days in their standard PCNL cohort [9]. 

### 3.2. Comparison of Post-Operative Outcomes

Additionally, post-operative outcomes appear to be similar or better in the tt-PCNL cohort compared to the standard PCNL cohort. The key post-operative outcomes tracked in this comparison are stone-free rates and post-operative pain. These post-operative outcomes are summarized in Table 2. Stone-free rate was reported by Yun et al. and Crook et al. Yun et al. reported a stone-free rate (based on either a post-op CT or KUB) of 73% in their standard PCNL cohort and 78% in their tt-PCNL cohort (*p* = 0.725) [9]. Crook et al. reported an intraoperative stone-free rate (assessed at the time of surgery) of 84% in the standard PCNL cohort and 96% in the tt-PCNL cohort [11]. 

Finally, in all cohorts except for Crook et al., there was improved pain control in the totally tubeless cohort when compared to t-PCNL or standard PCNL [7,8,9,10,11]. The assessment of pain varied between studies. All five studies compared post-operative analgesic dosage requirements to assess difference in pain and the tt-PCNL cohorts required less analgesia than their t-PCNL and standard PCNL cohort counterparts [7,8,9,10,11]. Bhat et al. also recorded subjective pain scores 24 h post-operatively using a “visual analog scale.” Based on this scale, Bhat reported the lowest pain score in the tt-PCNL cohort (3.04), followed by the t-PCNL cohort (4.76), and the worst pain score in the standard PCNL cohort (6.64) (*p* < 0.0001) [10].

### 3.3. Comparison of Complications

Major complications after PCNL are rare. In the literature, the reported major complication rate after PCNL is <5% [12]. After PCNL, the most common complications are urinary tract infection/sepsis, re-obstruction, and bleeding secondary to an arterio-venous malformation (AVM) [12,13]. Other, rarer complications are bowel injury, pneumothorax, urinoma, and urine leak [13]. The five comparative studies reported rates of complications. However, due to the rare nature of the complications and the low number of subjects in these studies, only Istanbulluoglu and Moosanejad reported significant data. The complications reported in these studies are summarized in Table 2.

In these two studies, they found no statistical difference in complications between tt-PCNL and standard PCNL and t-PCNL. In Istanbulluoglu et al.’s study, two patients in the tt-PCNL cohort, three in the t-PCNL cohort, and seven in the standard PCNL cohort developed complications (*p* = 0.783). Of note, two tt-PCNL patients had residual obstructing stones. One patient in the t-PCNL group and one in the standard PCNL group had an AVM requiring embolization. Whereas no tt-PCNL patients had fevers or UTIs, two t-PCNL and two standard PCNL patients had fevers or UTIs [8]. In Moosanejad et al.’s study, six patients in the standard PCNL group and four in the tt-PCNL group had complications (*p* = 0.73). Two patients in the standard PCNL and two in the tt-PCNL groups had fever. One patient in the standard PCNL group had an AVM, which was treated conservatively [8].

### 3.4. Other Considerations

#### 3.4.1. Ambulatory Totally Tubeless PCNL

Another important advancement in PCNL practice is the move from inpatient to ambulatory/outpatient PCNL. As described above, totally tubeless and tubeless PCNL reduce hospitalization length and require less pain relief, with no change in rates of bleeding or sepsis. This allows for select patients to safely undergo a PCNL in the ambulatory setting. While there is a limited amount of data available on ambulatory tt-PCNL, ambulatory t-PCNL has been well described. Chong and Davalos et al. reported their experience of 500 patients who underwent ambulatory PCNL in a free-standing surgery center. Of these patients, 496 were t-PCNL, 2 were tt-PCNL, and 2 were standard PCNL. In their cohort, only 12 patients required transfer from the ambulatory surgery center (2.4%) and their 30-day re-admission rate was 4.2% [14]. These data suggests that ambulatory tubeless PCNL is a safe and effective approach to performing PCNL in selected patients but that further research needs to be performed to assess the safety of tt- PCNL in the ambulatory setting. 

#### 3.4.2. Obesity and Totally Tubeless PCNL

Additionally, with rising rates of obesity, its impact on surgical outcomes is important to evaluate. Obesity is both a risk factor for stone formation and has been shown to impact overall surgical outcomes [15]. In the largest study to date, the Clinical Research Office of the Endourological Society (CROES) published a large series of PCNL procedures showing increased operative time, lower stone-free rates, and higher re-treatment rates in patients with a BMI > 40 [16]. In the largest comparison of PCNL technique in the obese population, Kuntz et al. evaluated 509 patients who underwent standard PCNL or t-PCNL and found no difference in stone free rate or complications based on BMI. Similarly, Yang et al. evaluated patients who underwent tubeless percutaneous renal surgery and found no difference in transfusion rate, length of stay or stone-free rate based on patient BMI [17]. Looking specifically at tt-PCNL, Aghamir et al. compared 78 obese patients who underwent either tt-PCNL or standard PCNL or t-PCNL and found similar operative times with lower analgesic use in the tt-PCNL cohort, which supports the use of totally tubeless technique in the obese population [18]. While the data are limited evaluating tt-PCNL, these data suggest that tt-PCNL is a safe and feasible option in obese patients. 

#### 3.4.3. Hemostatic Agents

As previously described, the two main reasons to leave a nephrostomy tube after PCNL is to achieve hemostasis and prevent the development of a urinoma [19]. With the development of tubeless and totally tubeless techniques for PCNL, different strategies have been developed to manage the nephrostomy tract. One option is to let the tract seal naturally and ensure adequate internal drainage. Another strategy is to place some form of hemostatic agent or sealant into the nephrostomy tract to augment closure. 

There are various sealants and hemostatic agents that have been described in the literature that operate using a variety of different mechanisms. These agents and their mechanisms are summarized in Table 3. FloSeal^®^ and Surgiflo^®^ are bovine and porcine gelatin matrices, Tisseel^®^ is a fibrin sealant, Spongostan^®^ is a gelatin sponge, and Surgicel^®^ is an oxidized cellulose matrix [20,21,22].

Multiple studies have evaluated the efficacy of using hemostatic agents after PCNL to help with hemostasis and prevention of urinoma. A meta-analysis by Yu et al. evaluated eight studies (six randomized control trials and two case control studies) comparing the usage of hemostatic agent versus no agent to seal the nephrostomy tract after PCNL [23]. The key parameters evaluated with length of stay, operative time, blood loss, transfusion rate, fever, and overall complication rate. Their analysis found that using a hemostatic agent was associated with a shorter overall length of stay but there was no difference in the other outcomes evaluated. A similar meta-analysis by Wang et al. reviewed seven studies comparing the usage of a hemostatic agent vs. control and found similar results to Yu. The meta-analysis evaluated length of stay, change in hemoglobin, blood transfusion, and analgesic requirement. Similarly, the only statistically significant difference was shorter hospital stay in the experimental group compared to control [24]. These two studies suggest that using a hemostatic agent or sealant is safe but that the only advantage is that it may lead to shorter hospital stays. While some authors suggest that sealants only add additional cost to the procedure, this is likely offset by the shorter overall hospital stay [24].

Choe et al. also describes early usage of electrocautery in the nephrostomy tract to prevent bleeding. While coagulative destruction in the nephrostomy tract may reduce bleeding, it may also prevent healing of the tract. This can create a nephro-cutaneous fistula or urinoma. Usage of a sealing agent not only allows for hemostatic closure of the tract but also provides a framework by which the renal parenchyma can begin to heal [25].

One possible risk of using a sealant or hemostatic agent is risk of obstruction of the urinary tract. If sealant is present in the urinary tract and does not dissolve, this could lead to post-operative obstruction. An in vitro study by Uribe et al. placed Surgicel^®^ (cellulose), FloSeal^®^ (gelatin matrix), Tisseel^®^ (fibrin sealant), and Coseal^®^ (polyethylene glycol) in contact with urine and blood to assess its reaction and solubility. The study found that Tisseel^®^, Coseal^®^, and Surgicel^®^ maintained a solid form upon initial contact with urine and blood that persisted for up to five days. However, the FloSeal^®^ gelatin matrix maintained a colloidal suspension and did not aggregate into a solid component. This suggests that gelatin-matrix-based hemostatic agents may be preferrable after percutaneous nephrolithotomy to prevent obstruction of the urinary tract if residual agent extravasates into the collecting system [26].

### 3.5. Technique

Various methods of totally tubeless strategy have been described. Totally tubeless PCNL can be performed with any body habitus, positioning approach, or access approach. The patient can be positioned in the supine or prone position and the surgeon can use fluoroscopy, ultrasound, or endoscopic combined intra-renal surgery to gain access. PCNL is performed via standard technique. Some authors leave the OR with no form of drainage, while other authors leave a ureteral catheter for up to 24 h post-operatively [7,8,9,10,11]. Our institution favors leaving it for only 1 h post-operatively as initially described by Aghamir et al. [27,28,29,30]. At our institution, patients are positioned in a modified Barts flank-free position. Cystoscopy is performed, followed by retrograde placement of a 0.035” hybrid wire in the renal pelvis, confirmed fluoroscopically. Ultrasound-guided access is established and confirmed fluoroscopically. Tract dilation is performed with a 17.5F Storz MIP-m set, ClearPetra 16F dilator and sheath, or with a 24F balloon dilator and sheath. Nephroscopy and lithotripsy are conducted with gravity irrigation using 3-L normal saline bags at a standardized height of 80 cm above the patient. At the conclusion of the case, a decision is made whether or not the patient can be left totally tubeless. Table 4 lists the criteria that need to be met for a patient to be left totally tubeless. Nephroscopy should show minimal ureteral/renal pelvic inflammation, no narrowing at the ureteropelvic junction, minimal intra-operative bleeding, and no residual fragments. Additionally, the patient should not have a solitary or functionally solitary kidney. If all these criteria are met, then the decision can be made to leave a patient totally tubeless.

An open-ended ureteral catheter is passed over a wire into the renal pelvis and the nephroscope is used to visualize proper placement of the end of the ureteral catheter in the renal pelvis. Once properly positioned, the nephroscope is removed and a hemostatic agent (FloSeal^®^) is applied within the nephrostomy tract. The sheath is removed and external pressure is applied to the kidney for two minutes to help with hemostasis. 

The ureteral catheter is left in place and tunneled into a foley catheter. The patient leaves the OR with the foley and ureteral catheter in place and goes to the recovery room. The foley catheter and ureteral catheter are left in place for one hour and then are removed prior to discharge from the post-anesthesia care unit. At this point, the patient is discharged totally tubeless. 

## 4. Discussion

In an effort to reduce morbidity from drainage catheters, there are a variety of drainage techniques employed by endourologists after nephrolithotomy. The historical standard is to leave a nephrostomy tube, which we refer to as standard PCNL [4]. Later, tubeless nephrolithotomy was developed, which refers to leaving some form of internalized drainage post-operatively (t-PCNL). This can include an indwelling double-J ureteral stent, an externalized ureteral catheter through the urethra, an externalized ureteral catheter through the flank, or some combination of these with a urethral foley catheter [4,31]. While some of these patients have their internalized tubes removed before discharge, they are not classified as totally tubeless if they are admitted for observation with some form of a drainage catheter. If a patient leaves the recovery room without any form internalized drainage (e.g., a foley catheter, ureteral stent with or without an antegrade or retrograde tether for removal, ureteral catheter, or nephrostomy tube), the procedure can then be truly considered to be totally tubeless nephrolithotomy (tt-PCNL) [5,31]. 

Totally tubeless PCNL was first introduced by Wickham et al., where tt-PCNL was performed when the “kidney was stone free with no excessive bleeding and had an intact collecting system” [32]. In 1986, there was an unsuccessful case series with totally tubeless PCNL by Winfield et al., where patients left tubeless had complications including increased pain, obstruction, urinary extravasation, and prolonged hospital stay [33]. Following their unsuccessful experience, there were several successful totally tubeless trials where tubeless patients had a decreased hospitalization stay, analgesia requirements, and an earlier return to normal activities with no change in complication, stone-free, or retreatment rates [27].

The five trials reviewed evaluated peri-operative outcomes, post-operative outcomes, and post-operative complications. These trials show that leaving a patient totally tubeless after PCNL has similar or better outcomes compared to leaving a stent or nephrostomy tube post-operatively. Looking at peri-operative outcomes, the trials reported no difference in blood loss or operative time. Only Moosanejad et al. report a statistically significant difference in operative time, but the difference was only 3 min, which is not clinically significant [7,8,9,10,11]. 

The studies reported two significant and likely related findings favoring the usage of totally tubeless technique—improved pain scores and shorter length of stay. Bhat, Yun, Moosanejad, and Istanbulluoglu all reported improved pain scores/analgesic requirements in the totally tubeless group [7,8,9]. This is likely explained by the lack of pain/discomfort caused by an internal or external tube (bladder spasms, stent colic, nephrostomy tube discomfort). These studies also report shorter length of stay for patients who were left totally tubeless. The shorter hospital stay is likely related to the improved pain scores in these patients. One alternative explanation is that patients left tubeless had less complex surgeries. However, Moosanejad, Istanbulluoglu, and Crook all compared pre-operative, operative, and stone characteristics and found no difference suggesting that the difference may not be attributed to differences in surgical complexity and may be better explained by decreased morbidity [7,8,11]. However, one limitation is that the studies are not blinded to the surgeon; the surgeon can ultimately decide whether or not to leave a tube. 

Two trials reported stone-free rates. Yun reported no difference in stone-free rates—73% in their standard PCNL cohort and 78% in their tt-PCNL cohort (*p* = 0.725). Crook reported a stone-free rate of 96% in their tt-PCNL cohort and 84% in their s-PCNL cohort (no statistics reported) [9,11]. The strategy for evaluating stone-free rates were different in these two studies. Yun et al. based the stone-free rates on post-operative imaging. Crooks et al. based their stone-free rate on intra-operative evaluation. While no statistics are reported, there is a large absolute 12% difference in stone-free rates in Crooks’ study. In this study, there was no differences in baseline characteristics and patients were only randomized if they were a candidate for totally tubeless technique. Since baseline characteristics and operative complexity should not have differed, one possible explanation for this difference is the lack of blinding to surgeons performing the intervention—surgeons may have been more likely to leave some fragments behind if they knew that the patient was randomized for nephrostomy tube placement. Yun et al.’s strategy for evaluating stone-free rates with post-operative imaging is more clinically relevant as most endourologists evaluate patients post-operatively with imaging to determine if any residual fragments are left. Importantly, this result highlights that leaving patients totally tubeless adequately drained any residual fragments and there was no difference in doing so compared to leaving a patient with a nephrostomy tube. 

The main concern for totally tubeless PCNL is lack of renal protection from obstruction post-operatively. Placing a stent or nephrostomy tube allows for drainage of urine, clots, and residual stone fragments, and a nephrostomy tube has the additional benefit of providing dilation tract hemostasis [4,5]. When performing a totally tubeless technique, the primary risk separating it from a stented PCNL is the risk of post-operative ureteral obstruction. In our review of the tt-PCNL literature, only Istanbulluoglu et al. reported on post-operative obstruction. They report that 2 of the 43 tt-PCNL patients developed post-operative obstruction (4.7%) [8]. As described above, there are many peri-operative benefits of the totally tubeless technique. However, it is important to perform this on carefully selected patients. 

A meta-analysis by Chen et al. evaluated 15 trials comparing tubeless PCNL to standard PCNL [34]. They found that tubeless PCNL reduced pain scores, analgesic requirements, and hospital stay without affecting blood loss and complication rate, supporting the use of tubeless PCNL over standard PCNL. Three of the studies in the present review (Yun et al., Moosanejad et al., and Crook et al.) compared totally tubeless PCNL to standard PCNL and also described that it reduced pain scores, analgesic requirements, and hospital stay without affecting blood loss or complications [7,9,11]. Similar to Chen et al.’s findings that support the use of tubeless PCNL over standard PCNL, these data support the use of totally tubeless PCNL over standard PCNL. Furthermore, Istanbulluoglu et al. and Bhat et al. compared totally tubeless PCNL to tubeless PCNL [8,10]. They both reported improved pain/analgesic requirements in the totally tubeless PCNL cohort. There was conflicting data regarding length of stay. Istanbulluoglu reported a slightly longer length of stay (2.09 for tt and 1.74 for t), while Bhat reported a slightly improved length of stay (2.48 days for tt and 3.20 for t) [8,10]. While more studies need to be performed to compare tubeless PCNL to totally tubeless PCNL, this suggests that totally tubeless PCNL not only improves outcomes compared to standard PCNL but also compared to tubeless PCNL.

Our practice favors totally tubeless technique when there is no inflammation or narrowing of ureteropelvic junction, no solitary kidney or functional solitary kidney, minimal intra-operative bleeding, and no concern for residual fragments at the conclusion of the procedure. 

In our technique, the positioning, access, and lithotripsy technique for totally tubeless PCNL does not differ from those of a standard PCNL or tubeless PCNL. Supine or prone positioning can be used during the operation and ultrasound or fluoroscopy can be used for access. The location of access can vary depending on the stone size, location, and surgeon preference. Our practice recommends balloon dilation to 24 French or less to minimize renal trauma and the surface area of the cut edge [35].

The purpose of leaving a tube, whether a nephrostomy or stent after PCNL, is to aid in drainage of residual stone fragments, blood clots, or infected urine [6]. Additionally, nephrostomy tube placement can help tamponade bleeding along the nephrostomy tract [6]. However, with the advent of minimally invasive tools, smaller dilation sheaths, and hemostatic agents, the risk of bleeding is lower [36,37]. Additionally, we have found that the usage of a ureteral occlusion balloon helps prevent fragments or blood clots from flowing antegrade down the ureter during PCNL. Finally, a thorough nephroscopy with a rigid nephroscope and a flexible cystoscope should be performed at the end of the procedure to identify and remove any residual stone fragments or large blood clots that could potentially cause obstruction [35,38].

Finally, when performing totally tubeless PCNL, our practice favors the usage of temporary ureteral drainage with an open-ended catheter. At the completion of the procedure, a 5 French open-ended catheter is placed into the collecting system, draining externally out of the body connected to a foley catheter. This temporarily allows for the kidney to drain after the procedure. The chief purpose of this catheter is to assure that the collecting system remains collapsed, promoting closure of the percutaneous tract. By keeping the collecting system empty, this also helps prevent any large clots from forming. This catheter remains in place for one hour and is removed with the foley catheter in the recovery room before discharge. 

While the current review favors the usage of totally tubeless percutaneous nephrolithotomy, the current literature is not without limitations. Some studies are retrospective in nature so it is challenging to control for intra-operative factors that may have favored leaving a patient totally tubeless, which also may have explained why their outcomes are improved. Additionally, in the studies that were randomized trials, it is impossible to blind the surgeon to the treatment option, which also could confound the peri-operative and post-operative outcomes reported. In the future, larger randomized control trials comparing outcomes of outpatient tubeless PCNL with a stent and tt-PCNL would provide further evidence of its feasibility, safety, and efficacy.

## 5. Conclusions

Totally tubeless percutaneous nephrolithotomy is a procedure aimed at reducing morbidity associated with the procedure. The current literature suggests that leaving patients totally tubeless after percutaneous nephrolithotomy does, indeed, reduce morbidity without compromising outcomes. Ultimately, leaving a patient totally tubeless led to less pain and shorter hospital stays while not affecting stone-free rates or bleeding risk post-operatively. Shorter hospital stays and less pain not only improves patient outcomes but also reduces cost burden to the patients and the healthcare system. However, a multicentered prospective randomized control trial needs to be performed in order to directly compare tt-PCNL to t-PCNL. 

## Figures and Tables

**Table 1:** Study methodology. Comparison groups and post-operative protocols.

Methods of Studies							
			Comparitive Groups		Specific Techniques		
Manuscript	Authors	Population (n)	Intervention	Control	OR Sheath and Dilation	Intervention	Control
Comparative Study between Standard and Totally Tubeless Percutaneous Nephrolithotomy	Yun et al. [9]	57	tt-PCNL	s-PCNL	28fr sheath Balloon dilation	Totally Tubeless. No tubes post-operatively.	20fr malecot drain left as a nephrostomy tube for 3–5 days post-operatively
Comparison of Totally Tubeless Percutaneous Nephrolithotomy and Standard Percutaneous Nephrolithotomy for Kidney Stones: A Randomized, Clinical Trial	Moosanejad et al. [7]	84	tt-PCNL	s-PCNL	28–30fr sheath Amplatz dilators	Totally Tubeless. Temporary ureteral and foley catheter in recovery removed after 4 h.	Nephrostomy tube (size not mentioned) and 4fr or 5fr ureteral catheter.
A Randomized Controlled Study Comparing the Standard, Tubeless, and Totally Tubeless Percutaneous Nephrolithotomy Procedures for Renal Stones From a Tertiary Care Hospital	Bhat et al. [10]	75	tt-PCNL	s-PCNL and t-PCNL	30fr dilation (sheath not mentioned) Amplatz dilators	Totally Tubeless. No specific tubes in recovery mentioned.	s-PCNL: 22fr nephrostomy tube t-PCNL: indwelling ureteral stent
A Randomized Controlled Trial of Nephrostomy Placement Versus Tubeless Percutaneous Nephrolithotomy	Crook et al. [11]	50	tt-PCNL	s-PCNL	Dilation and sheath not mentioned	Totally Tubeless. No tubes post-operatively.	26fr nephrostomy tube left postoperatively and removed on day of discharge
Percutaneous Nephrolithotomy: Nephrostomy or Tubeless or Totally Tubeless?	Istanbulluoglu et al. [8]	176	tt-PCNL	s-PCNL and t-PCNL	30fr sheath Amplatz dilators	Totally tubeless. Temporary ureteral catheter removed immediately post-operatively	s-PCNL: 14fr nephrostomy tube t-PCNL: indwelling ureteral stent

**Table 2:** Summary of trials reviewed comparing standard, tubeless, and totally tubeless percutaneous nephrolithotomy. tt = totally tubeless PCNL, t = tubeless PCNL, s = standard PCNL.

Results of Studies											
		Methods	Perioperative Outcomes			Postoperative Outcomes		Complications			
Manuscript	Authors	Comparison	Op Time	Blood Loss	Hospital Stay (Days)	Pain	Stone Free Rate	Overall Complications	Fever/UTI	Obstruction	AVM
Comparative Study between Standard and Totally Tubeless Percutaneous Nephrolithotomy	Yun et al. [9]	s-PCNL vs. tt-PCNL	tt = s	Same	tt < s 3.92 vs. 8.25	tt > s tt better	tt = s 77.8% vs. 73.3%	N/A	1 tt-PCNL 1 s-PCNL	N/A	1 s-PCNL
Comparison of Totally Tubeless Percutaneous Nephrolithotomy and Standard Percutaneous Nephrolithotomy for Kidney Stones: A Randomized, Clinical Trial	Moosanejad et al. [7]	s-PCNL vs. tt-PCNL	tt < s	Same	tt < s 1.25 vs. 2.95	tt-PCNL better (assessed pethidine requirement)	N/A	Same	2 tt-PCNL 2 s-PCNL	N/A	1 s-PCNL
A Randomized Controlled Study Comparing the Standard, Tubeless, and Totally Tubeless Percutaneous Nephrolithotomy Procedures for Renal Stones From a Tertiary Care Hospital	Bhat et al. [10]	s-PCNL vs. t-PCNL vs. tt-PCNL	N/A	Same	tt < t < s 2.48 vs. 3.20 vs. 4.16	tt > t > s tt best	N/A	N/A	3 t-PCNL 2 s-PCNL	N/A	N/A
A Randomized Controlled Trial of Nephrostomy Placement Versus Tubeless Percutaneous Nephrolithotomy	Crook et al. [11]	s-PCNL vs. tt-PCNL	N/A	Same	tt < s 2.32 vs. 3.36	Same statistically but tt-PCNL trended less	96%(tt) vs. 84%(s) (statistics not reported)	N/A	2 tt-PCNL 2 s-PCNL	N/A	N/A
Percutaneous Nephrolithotomy: Nephrostomy or Tubeless or Totally Tubeless?	Istanbulluoglu et al. [8]	s-PCNL vs. t-PCNL vs. tt-PCNL	tt = t = s	Same	tt > t < s 2.09 vs. 1.74 vs. 2.96	Same NSAIDS requirement tt-PCNL and t-PCNL required less narcotics	N/A	Same	2 t-PCNL 2 s-PCNL	2/43 pts in the tt-PCNL group obstructed	1 t-PCNL 1 s-PCNL

**Table 3:** Sealant and hemostatic agents used after PCNL.

Agent	Mechanism
FloSeal^®^	Bovine Gelatin Matrix
Surgiflo^®^	Porcine Gelatin Matrix
Tisseel^®^	Fibrin Sealant
Spongostan^®^	Absorbable hemostatic gelatin sponge
Surgicel^®^	Oxidized Cellulose Matrix

**Table 4:** Criteria for leaving a patient totally tubeless after PCNL.

Criteria for Totally Tubeless Nephrolithotomy
Minimal renal/ureteral inflammation
No narrowing at ureteropelvic junction
No solitary or functionally solitary kidney
Minimal Intra-operative bleeding
No residual fragments
